# Adherence to the Mediterranean diet and female sexual function: a scoping review

**DOI:** 10.1093/sexmed/qfag098

**Published:** 2026-10-26

**Authors:** Aysu Yildiz Karaahmet, Fatma Şule Bilgiç

**Affiliations:** Biruni University Faculty of Health Sciences, Department of Midwifery, Istanbul 34100, Turkey; Çanakkale Onsekiz Mart University Faculty of Health Sciences, Department of Midwifery, Çanakkale 17100, Turkey

**Keywords:** Mediterranean diet, female sexual dysfunction, female sexual function, female sexual health, women’s health, scoping review

## Abstract

**Objective:**

This scoping review aimed to systematically map the available evidence on the association between Mediterranean diet adherence and female sexual health and to explore potential biological and psychological mechanisms underlying this relationship.

**Methods:**

The review was conducted according to the methodological frameworks of Arksey and O’Malley, Levac et al., and the Joanna Briggs Institute. MEDLINE, Scopus, Web of Science, Embase, PsycINFO, and CINAHL were systematically searched, supplemented by manual reference screening. Reporting followed the PRISMA-ScR guideline, and methodological quality was assessed using the Mixed Methods Appraisal Tool (MMAT).

**Results:**

Seven studies met the inclusion criteria. Overall, higher Mediterranean diet adherence was associated with more favorable female sexual health outcomes. Greater adherence was linked to lower rates of female sexual dysfunction and higher Female Sexual Function Index scores among women with type 2 diabetes; reduced deterioration in sexual function in randomized evidence; and improved lubrication, orgasm, and sexual satisfaction among women with severe obesity undergoing bariatric surgery. Favorable associations were also reported in women with inflammatory bowel disease and endometriosis, whereas in chronic liver disease, the initially observed associations were no longer statistically significant after multivariable adjustment. Proposed mechanisms included improved vascular and metabolic health, reduced inflammation and oxidative stress, and enhanced psychological well-being.

**Conclusion:**

Higher Mediterranean diet adherence was generally associated with more favorable female sexual function and well-being; however, the predominantly observational and cross-sectional nature of the evidence precludes causal inference. The limited evidence base highlights the need for high-quality longitudinal and interventional studies.

## Introduction

Female sexual health is a fundamental determinant of individual well-being and quality of life and is recognized as an important component of physical, psychological, and social health. A healthy sexual life is closely associated with life satisfaction, interpersonal relationships, perceived health status, and overall well-being.^1^ Female sexual function is a complex and multifactorial process sustained through the coordinated interaction of neurological, vascular, hormonal, and psychosocial mechanisms. Disruptions in any of these systems may adversely affect the female sexual response cycle and contribute to sexual dysfunction.^1^^,^^2^

The World Health Organization (WHO) defines sexual health not merely as the absence of disease or dysfunction but as a state of physical, emotional, mental, and social well-being related to sexuality.^3^ Accordingly, female sexuality is increasingly recognized as a multidimensional construct shaped by the interaction of biological, psychological, relational, and sociocultural factors.^4^ Historically, however, scientific understanding of female sexuality developed relatively late, resulting in female sexual health being viewed primarily through reproductive or psychological frameworks. Over recent decades, female-centered models have highlighted that female sexual function is closely linked not only to psychosocial factors but also to vascular, neuroendocrine, and metabolic processes.^1^^,^^5^

Female sexual dysfunction (FSD) is a prevalent public health concern affecting a substantial proportion of women worldwide. Meta-analytic evidence suggests that approximately 40% of adult women report at least 1 symptom of sexual dysfunction, based mainly on cross-sectional studies using self-report instruments; accordingly, this figure should be interpreted as a pooled prevalence estimate rather than a verified lifetime risk.^2^ Its prevalence is particularly elevated among women with diabetes, hypertension, obesity, metabolic syndrome, and cardiovascular disease. Chronic inflammation, endothelial dysfunction, and vascular impairment have also been shown to negatively affect genital blood flow, thereby compromising arousal, lubrication, and orgasmic function.^6^ These findings indicate that female sexual health cannot be explained solely by psychological mechanisms and that vascular and metabolic pathways play a central role.

Current management strategies for FSD primarily focus on psychoeducation, cognitive behavioral therapy, and couples-based interventions.^7^ Nevertheless, growing evidence suggests that dietary and lifestyle factors may be associated with female sexual health. Because obesity, insulin resistance, and chronic inflammation are modifiable through dietary interventions, nutrition-based approaches have been proposed as potentially supportive approaches for female sexual health outcomes.^2^^,^^8^^,^^9^

Among dietary patterns, the Mediterranean diet has received particular attention because of its anti-inflammatory, antioxidant, and cardioprotective properties. Rather than a set of individual foods, the Mediterranean diet is best characterized as an overall dietary pattern, with olive oil as the principal source of dietary fat; a high intake of fruits, vegetables, whole grains, legumes, and nuts; moderate fish and dairy intake; and limited red and processed meat. This pattern has been associated with improved endothelial function, greater nitric oxide bioavailability, and better vascular health, all of which may positively influence genital blood flow and sexual function.^6^ Furthermore, healthy dietary patterns may contribute to hormonal balance, reduce systemic inflammation, and improve psychological well-being.^10^

Given the close relationship between vascular health and nutrition, dietary patterns that promote nitric oxide production and reduce inflammation may be relevant to female sexual health. Indeed, recent systematic reviews have reported significant improvements in female sexual function among women adhering to healthy dietary patterns.^9^ In addition, the beneficial effects of healthy diets on depression and anxiety symptoms suggest that nutrition may influence female sexual health through interconnected biological and psychosocial pathways.^2^

Despite growing interest in this field, the literature examining the relationship between nutrition and female sexual health remains fragmented. Evidence specifically addressing adherence to the Mediterranean diet and female sexual function is dispersed across different populations, assessment tools, and methodological approaches, resulting in considerable heterogeneity. Previous reviews have addressed narrower questions: The effects of healthy dietary patterns in general on sexual function,^9^ lifestyle determinants of sexuality in both sexes,^10^ and the influence of the Mediterranean pattern on male sexual dysfunction and FSD from a mainly clinical perspective.^2^ None of them has mapped, across study designs and clinical populations, which domains of female sexual health have been studied, which measurement instruments have been used, and where the evidence gaps lie. A scoping review is therefore the most appropriate design, as the purpose is to map the extent, range, and nature of the available evidence and to identify gaps rather than to estimate a pooled effect. Accordingly, the aim of this scoping review was to systematically map the existing literature on the association between adherence to the Mediterranean diet and female sexual function, characterize study designs, populations, and assessment instruments, map the biological and psychosocial mechanisms proposed in the literature, and identify gaps to guide future research.

## Method

In line with the objectives of this study, a scoping review methodology was selected to systematically map the existing literature on the relationship between adherence to the Mediterranean diet and female sexual health outcomes, identify knowledge gaps, and provide directions for future research.^11^

The available evidence is methodologically heterogeneous with respect to study populations, dietary assessment methods, and female sexual health outcomes. Therefore, a scoping review was considered more appropriate than a conventional systematic review or meta-analysis for comprehensively mapping the existing evidence.

This review was conducted according to the methodological framework proposed by Arksey and O’Malley,^12^ further refined by Levac et al.,^13^ and guided by the recommendations of the Joanna Briggs Institute (JBI).^14^ Reporting followed the Preferred Reporting Items for Systematic Reviews and Meta-Analyses Extension for Scoping Reviews (PRISMA-ScR) guidelines, and the PRISMA-ScR checklist is provided as supplementary material.

The review process comprised the following stages: (1) defining the research questions; (2) developing the search strategy; (3) identifying relevant studies; (4) selecting eligible studies; (5) extracting and charting data; (6) descriptively assessing the methodological characteristics of the included studies; and (7) summarizing the evidence and identifying knowledge gaps.

### Research questions

This comprehensive review aims to systematically map the existing literature on the relationship between the Mediterranean diet and female sexual function and sexual health outcomes, seeking answers to the following research questions:

1. What is the current evidence regarding the association between adherence to the Mediterranean diet and female sexual health outcomes?
2. Which domains of female sexual health (eg, sexual desire, arousal, lubrication, orgasm, satisfaction, pain, and overall sexual function) have been reported in association with adherence to the Mediterranean diet?
3. Which biological, metabolic, and psychosocial mechanisms linking the Mediterranean diet and female sexual health are proposed in the existing literature?
4. How does the association between the Mediterranean diet and female sexual health differ across clinical populations (eg, type 2 diabetes, obesity, inflammatory bowel disease, chronic liver disease, endometriosis, and the general female population)?
5. What methodological approaches, measurement tools, and evidence gaps characterize the existing literature on this topic?

### Search strategy and data sources

The literature search was conducted between 1 January and 1 February 2026, without restrictions on publication year. For each database, the final search date, the complete search string, the filters applied (English language; human studies), and the number of records retrieved are reported in Table 1, so that the searches can be reproduced. Electronic searches were performed in MEDLINE (via PubMed), Embase, Scopus, Web of Science Core Collection, PsycINFO, and CINAHL (Cumulative Index to Nursing and Allied Health Literature) to identify studies examining the association between adherence to the Mediterranean diet and female sexual health outcomes.

**Table 1:** Databases searched and search strategy.

**Database**	**Date searched**	**Search terms (concept blocks)**	**Limits/filters**
MEDLINE (via PubMed)	January 1-February 1, 2026	Population: women, female^*^, adult women, menopausal women, women with chronic disease Exposure: “Mediterranean Diet” MeSH, Mediterranean diet, dietary pattern^*^, diet quality, Mediterranean dietary adherence, nutrition, dietary habits Outcome: “Sexual Health” MeSH, sexual function, female sexual dysfunction, FSFI, libido, sexual desire, sexual satisfaction, sexual well-being, sexual distress, dyspareunia, orgasm, lubrication Mechanisms/related factors: inflammation, oxidative stress, metabolic syndrome, obesity, diabetes, menopause, quality of life	English language; human studies
Embase	January 1-February 1, 2026	female/women AND Mediterranean diet/dietary pattern/diet quality AND sexual function/female sexual dysfunction/libido/sexual health/FSFI AND inflammation/oxidative stress/metabolic syndrome	English language; human studies
Scopus	January 1-February 1, 2026	women AND (Mediterranean diet OR dietary adherence) AND (sexual function OR female sexual dysfunction OR libido OR sexual well-being OR FSFI) AND (inflammation OR quality of life OR metabolic health)	English language
Web of Science Core Collection	January 1-February 1, 2026	TS = ((women OR female^*^) AND (“Mediterranean diet” OR dietary adherence) AND (“sexual function” OR “female sexual dysfunction” OR libido OR “sexual health” OR FSFI OR dyspareunia OR sexual satisfaction))	English language
PsycINFO	January 1-February 1 2026	(women OR female^*^) AND (Mediterranean diet OR healthy diet) AND (sexual well-being OR sexual satisfaction OR sexual distress OR quality of life)	English language
CINAHL (EBSCOhost)	January 1-February 1, 2026	(women OR female^*^) AND Mediterranean diet AND (sexual function OR female sexual dysfunction OR sexual health OR sexual satisfaction OR dyspareunia)	English language
Grey Literature	January 1-February 1, 2026	OpenGrey; ProQuest Dissertations & Theses; manual screening of reference lists of included studies.	English language

The search strategy combined controlled vocabulary (MeSH and database-specific subject headings) and free-text terms related to the Mediterranean diet (eg, Mediterranean diet, Mediterranean dietary pattern, and Mediterranean diet adherence) and female sexual health (eg, female sexual function, FSD, sexual health, sexual satisfaction, sexual well-being, and dyspareunia). Boolean operators (AND/OR), truncation, and database-specific adaptations were applied as appropriate. To ensure comprehensive retrieval, terms describing both Mediterranean diet adherence and multidimensional female sexual health outcomes were included. The complete search strategy for each database is presented in Table 1.

### Grey literature review and manual search

In addition to published literature, a gray literature review was also conducted. This included searching the OpenGrey and ProQuest Dissertations & Theses databases; master’s and doctoral theses focusing particularly on the Mediterranean diet, women’s health, sexual function, and lifestyle-related health outcomes were examined. The main objective of the gray literature review was to identify unpublished studies that could contribute conceptually or methodologically. Furthermore, journals closely related to the topic (eg, journals in the fields of sexual health, women’s health, nutrition, metabolic health, and lifestyle medicine) were manually searched; the bibliographies of the included studies were reviewed to identify additional studies that might have been overlooked in the database searches. This process was repeated until no new suitable studies were found. Because eligibility was restricted to peer-reviewed articles published in English, theses and other unpublished documents were not eligible for inclusion in the synthesis; the gray literature search was therefore used only to identify additional peer-reviewed publications and to check whether any conceptual or methodological contribution had been overlooked. No gray literature source was included among the seven studies synthesized.

The titles and abstracts of all potentially relevant studies were independently screened by 2 reviewers (A.Y.K. and F.Ş.B.). Subsequently, full-text eligibility assessments were also performed independently by the same 2 reviewers. Any disagreements regarding study eligibility were resolved through discussion until consensus was reached. The study selection process is reported in detail using the PRISMA-ScR flowchart (Figure 1).

**Figure 1:**
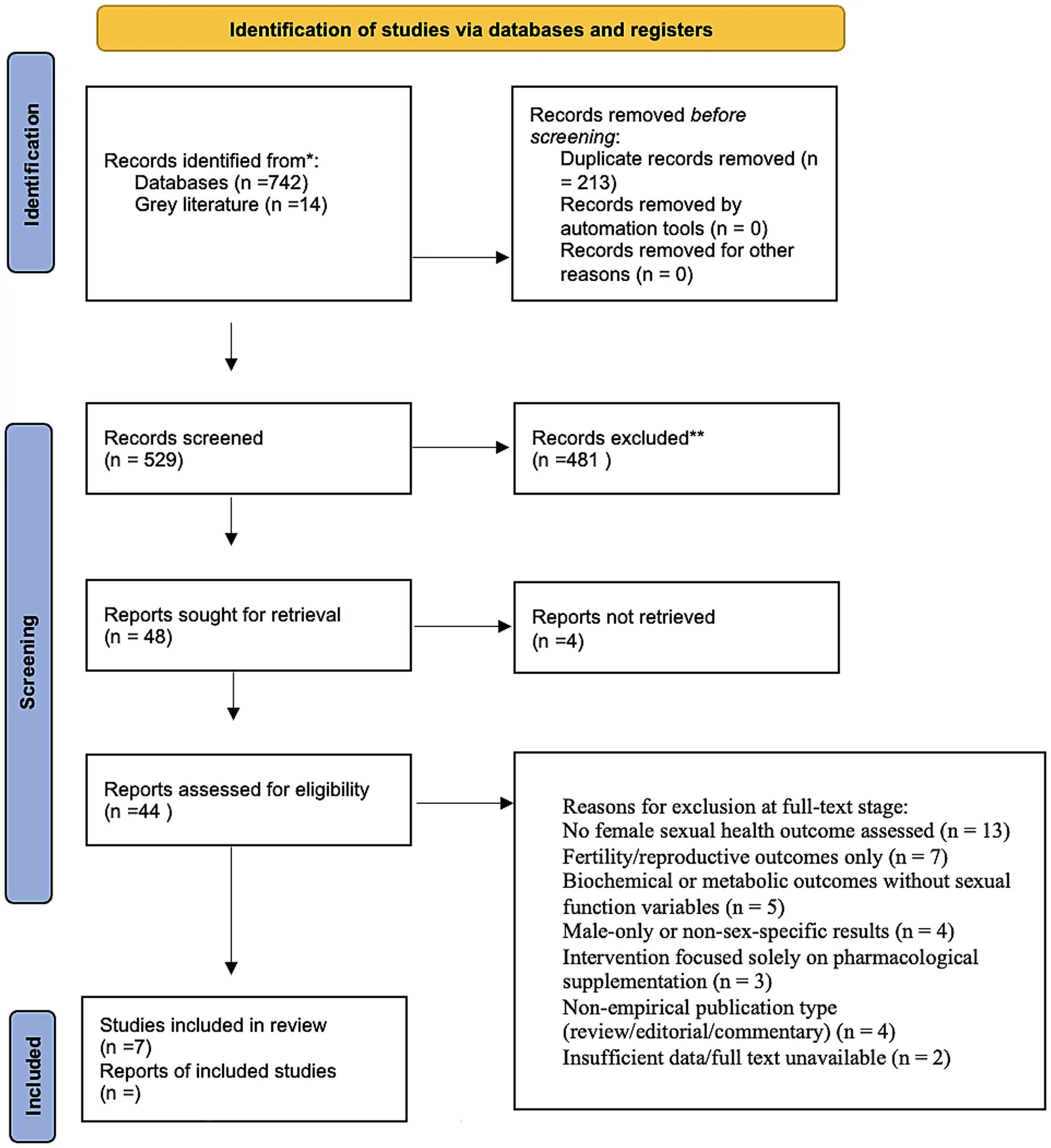
PRISMA-ScR flow diagram illustrating the study selection process.

### Inclusion and exclusion criteria

#### Inclusion criteria

Studies were included if they:

- Included female participants or reported female-specific outcomes separately;
- Evaluated the Mediterranean diet, Mediterranean diet adherence, Mediterranean dietary pattern, or Mediterranean diet–based interventions as the primary exposure or intervention;
- Assessed at least 1 female sexual health outcome (eg, sexual function, sexual dysfunction, sexual satisfaction, sexual well-being, sexual desire, dyspareunia, or related outcomes);
- Reported female sexual health as a primary or secondary outcome;
- Employed an observational, experimental, quasi-experimental, or mixed-methods design;
- Were published in peer-reviewed journals in English.

#### Exclusion criteria

Studies were excluded if they:

- Did not evaluate the Mediterranean diet as the primary exposure;
- Focused exclusively on fertility; reproductive outcomes; or biochemical, hormonal, or metabolic parameters without assessing female sexual health outcomes;
- Included only male participants or did not report female-specific outcomes separately;
- Focused exclusively on male sexual function;
- Evaluated only isolated dietary components, supplements, or pharmacological interventions rather than the overall Mediterranean dietary pattern;
- Were reviews, editorials, conference abstracts, letters, commentaries, or animal studies; or
- Had unavailable full texts or were published in languages other than English.

Because terminology related to female sexual health varies across the literature, eligibility was determined according to the context of each study rather than a single predefined outcome measure. The inclusion and exclusion criteria were applied iteratively in accordance with scoping review methodology to ensure conceptual consistency. Iterative application refers solely to the consistent interpretation of the predefined criteria as familiarity with the literature increased; no eligibility criterion was added, removed, or modified at any stage of the screening process, and no post hoc change to the criteria influenced study selection.

### Study selection

All records were transferred to a reference management software, and duplicate records were removed. Then, the titles and abstracts of the studies were systematically screened according to predetermined inclusion and exclusion criteria. Studies examining the relationship between the Mediterranean diet, adherence to the Mediterranean diet, or Mediterranean diet–based interventions and female sexual health outcomes were considered potentially suitable.

Following the preliminary evaluation, the full texts of the studies deemed suitable were reviewed in detail, and the final inclusion decision was made. When evaluating the suitability of studies, those examining the relationship between exposure to or adherence to the Mediterranean diet and female sexual health outcomes (eg, libido/sexual desire, female sexual function, arousal, vaginal lubrication, orgasm, sexual satisfaction, sexual well-being, dyspareunia, or FSD) were considered. In accordance with the scoping review methodology, the study selection process was conducted iteratively; as familiarity with the literature increased, inclusion decisions were reviewed as needed to maintain conceptual consistency. The study selection process is reported in detail using the PRISMA-ScR flowchart (Figure 1).

### Data extraction and charting

A standardized data extraction form was developed to ensure consistent and systematic data collection. For each included study, the following information was extracted: study characteristics (author, year, country, study design, and participant characteristics), Mediterranean diet exposure or intervention, female sexual health outcomes and assessment methods, proposed biological or clinical mechanisms, and key findings and identified knowledge gaps. Data extraction was conducted systematically and iteratively, and any conceptual differences identified during data charting were reviewed within the context of each study to ensure consistency. Data extraction was performed independently by 2 reviewers (A.Y.K. and F.Ş.B.) using the same form; the extracted data were subsequently compared item by item, disagreements were resolved through discussion, and a third reviewer was consulted when consensus could not be reached.

### Quality assessment

Although formal critical appraisal is not mandatory in scoping reviews, a descriptive methodological assessment was conducted using the 2018 version of the Mixed Methods Appraisal Tool (MMAT) to characterize the methodological quality of the included studies. The purpose of this assessment was not to exclude studies or rank the level of evidence but rather to contextualize the strengths and limitations of the available literature. The MMAT was applied independently by 2 reviewers, and discrepancies were resolved by discussion. For each study, the design-specific criteria corresponding to its methodology (randomized controlled trials, non-randomized studies, and quantitative descriptive studies) were applied. In line with the developers’ recommendation, no overall numerical quality score was calculated; instead, responses to each criterion are presented item.

The assessment considered key methodological characteristics, including study design, sample characteristics, dietary assessment methods, outcome measurement tools, and clinical heterogeneity across studies. Particular attention was given to differences in the assessment of Mediterranean diet adherence and female sexual health outcomes, as well as the predominance of cross-sectional designs. The findings of the quality assessment were used to support the interpretation of the evidence base rather than as a basis for study exclusion.

## Results

### Study selection

A total of 742 records were identified through database and manual searches. After duplicate removal, 529 records were screened by title and abstract. Of these, 48 full-text articles were assessed for eligibility; 4 could not be retrieved, leaving 44 articles for full-text review. Ultimately, 7 studies met the predefined inclusion criteria and were included in this scoping review (Figure 1). The number of records at each stage and the reasons for exclusion listed below correspond exactly to the PRISMA-ScR flow diagram: of the 44 full texts reviewed, 37 were excluded and 7 were included.

Studies were excluded for the following reasons: failure to report female sexual health outcomes despite evaluating dietary patterns (n = 12); inclusion of only male participants or failure to report female-specific outcomes separately (n = 4); focus on fertility, reproductive outcomes, menopausal symptoms, pelvic pain, or general quality of life without directly assessing female sexual function (n = 7); assessment of only biochemical, hormonal, or metabolic outcomes (n = 5); evaluation of pharmacological or supplement-based interventions (n = 3); non-empirical publication types (n = 4); and unavailable full text or insufficient data (n = 2).

However, some studies addressing broader aspects of women’s health and sexuality were not included in the main synthesis but were considered as contextual evidence. For example, the cross-sectional study by Haghshenas et al.^22^ examined the association between adherence to the Mediterranean diet and menopause-related symptoms and quality of life. Because it did not directly evaluate female sexual function or FSD using a validated sexual function measure, it did not meet the predefined inclusion criteria and was therefore considered contextual evidence rather than an included study (Table 2). Accordingly, the menopause-related findings of this study are reported only as background information and are not part of the synthesis of the 7 included studies. The study selection process was conducted in accordance with PRISMA-ScR recommendations, and a detailed flowchart is presented in (Figure 1).

**Table 2:** Inclusion and exclusion criteria.

**Category**	**Inclusion criteria**	**Exclusion criteria**
**Population**	Women or female participants; studies reporting female-specific outcomes separately.	Studies including only men or mixed-sex populations without sex-specific analyses.
**Exposure**	Mediterranean diet adherence, Mediterranean dietary pattern, Mediterranean diet intervention, or diet quality related to Mediterranean dietary habits.	Studies focusing exclusively on non-Mediterranean dietary exposures without Mediterranean diet assessment.
**Outcome of Interest**	Female sexual health outcomes, including sexual function, female sexual dysfunction, libido/sexual desire, arousal, lubrication, orgasm, dyspareunia, sexual satisfaction, sexual well-being, sexual distress, or FSFI-related outcomes.	Studies focusing exclusively on fertility, ovulation, reproductive outcomes, or non-sexual health outcomes without assessment of female sexual health.
**Related Mechanisms/Health Domains**	Biological, psychological, or behavioral factors related to female sexual health (eg, inflammation, oxidative stress, metabolic health, obesity, diabetes, menopause, quality of life, pain burden).	Studies evaluating only biochemical, hormonal, or metabolic parameters without female sexual health outcomes.
**Study Design**	Quantitative, qualitative, mixed-methods studies, observational studies, clinical trials, and prospective interventions.	Editorials, opinion papers, conference abstracts, letters to editors, case reports, narrative reviews, and non-empirical publications.
**Language**	English.	Non-English publications.
**Publication Type**	Peer-reviewed journal articles.	Non–peer-reviewed publications.
**Time Frame**	No restriction on publication year.	—

Abbreviation: FSFI, Female Sexual Function Index.

### Characteristics of included studies

The characteristics of the included studies are summarized in Table 3. Overall, the literature examining the relationship between the Mediterranean diet and female sexual health remains limited and methodologically heterogeneous. Most studies were quantitative observational investigations, predominantly cross-sectional in design, while a smaller number consisted of randomized controlled trials, prospective intervention studies, and longitudinal investigations.^15–17^

**Table 3:** Characteristics of included studies.

**Author (year)**	**Country**	**Study design**	**Population/sample**	**Mediterranean diet assessment**	**Sexual health measure**	**Key findings**
Giugliano et al.^15^	Italy	Cross-sectional observational study	Women with type 2 diabetes (n = 595)	9-Point Mediterranean Diet Score	FSFI	Greater adherence to the Mediterranean diet was associated with higher sexual activity and lower prevalence of female sexual dysfunction (FSD).
Maiorino et al.^16^	Italy	Randomized controlled trial (MÈDITA trial)	Women with newly diagnosed type 2 diabetes (approx. n = 109)	Mediterranean diet intervention vs. low-fat diet	FSFI	Women in the Mediterranean diet group showed a smaller decline in sexual function over long-term follow-up compared with the low-fat diet group.
Ruiz-Tovar et al.^17^	Spain	Retrospective observational study	Women with severe obesity undergoing bariatric surgery (n = 240)	PREDIMED adherence questionnaire	FSFI	Better postoperative adherence to the Mediterranean diet was associated with higher lubrication, orgasm, and satisfaction scores.
Romano et al.^18^	Italy	Cross-sectional observational study	Women with inflammatory bowel disease (n = 134)	Mediterranean Diet Score	FSFI	Higher Mediterranean diet adherence was independently associated with lower odds of female sexual dysfunction (OR = 0.80, 95% CI 0.653-0.974).
Romano et al.^19^	Italy	Cross-sectional observational study	Women with chronic liver disease (n = 84)	Mediterranean Diet Score	FSFI	Mediterranean diet adherence showed positive associations in univariate analyses but was not an independent protective factor for sexual dysfunction after multivariate adjustment.
Cirillo et al.^20^	Italy	Prospective intervention study	Women with endometriosis (n = 35; n = 26 completed follow-up)	Mediterranean diet intervention	VAS for dyspareunia and pelvic pain	Increased Mediterranean diet adherence was associated with reductions in dyspareunia and chronic pelvic pain symptoms.
Ott et al.^21^	Austria	Observational intervention study	Women with endometriosis	Mediterranean diet recommendation	Pain and symptom assessment	Mediterranean diet recommendation was associated with improvements in endometriosis-related symptoms and pain.

Abbreviations: FSFI, Female Sexual Function Index; VAS, Visual Analog Scale.

The majority of studies were conducted in European countries, particularly Italy and Spain, likely reflecting the widespread adoption of the Mediterranean diet as a traditional dietary pattern in these regions. Most investigations focused on clinical populations, including women with type 2 diabetes mellitus (T2DM), inflammatory bowel disease (IBD), morbid obesity, chronic liver disease, and endometriosis.^15–18^ This suggests that the current evidence base is largely derived from women with chronic health conditions rather than from the general population.

Participant characteristics varied considerably across studies. Research involving women with T2DM primarily included middle-aged adults, with sample sizes ranging from approximately 100 to 600 participants.^15^^,^^16^ The bariatric surgery study included heterosexual women with severe obesity,^17^ whereas studies on IBD and chronic liver disease enrolled both male and female participants but reported female sexual health outcomes separately.^19^ The prospective study conducted among women with endometriosis focused on women of reproductive age.^20^^,^^21^

Methodologically, cross-sectional studies represented the most common design and primarily examined associations between Mediterranean diet adherence and female sexual function. Dietary adherence was generally assessed using validated Mediterranean diet adherence scores, while the Female Sexual Function Index (FSFI) was the most frequently used instrument for evaluating sexual health outcomes.^15–17^^,^^19^ Several studies also examined FSFI subdomains, including desire, arousal, lubrication, orgasm, satisfaction, and pain.

Observational studies generally reported that greater adherence to the Mediterranean diet was associated with more favorable sexual health outcomes. Among women with T2DM, higher adherence was linked to lower prevalence of FSD and higher FSFI scores.^15^ Similarly, adherence to the Mediterranean diet was inversely associated with FSD in women with IBD. However, among women with well-compensated chronic liver disease, initially positive associations did not remain significant after multivariable adjustment.^18^

Although fewer in number, intervention studies provided evidence from prospective designs. In the long-term MÈDITA randomized controlled trial, women assigned to a Mediterranean diet experienced less deterioration in sexual function over time and demonstrated more favorable FSFI outcomes compared with those following a low-fat diet.^16^ Likewise, among women undergoing bariatric surgery, greater adherence to the Mediterranean diet was associated with higher scores in lubrication, orgasm, and sexual satisfaction domains.^17^

Sexual health outcomes extended beyond traditional measures of sexual function. In women with endometriosis, increased adherence to the Mediterranean diet was associated with reductions in dyspareunia, pelvic pain, and related pain symptoms,^20^ suggesting that the diet may influence not only sexual function but also symptom burden and overall sexual well-being.

The relationship between the Mediterranean diet and female sexual health was generally explained through both biological and psychosocial pathways. Proposed mechanisms included improvements in glycemic control and metabolic health, enhanced endothelial and vascular function, reductions in inflammation and oxidative stress, hormonal regulation, decreased depressive symptoms, and improved quality of life.^15^^,^^16^^,^^20^ These mechanisms may be particularly relevant in women with diabetes, obesity, and chronic inflammatory conditions.

From a methodological perspective, the use of validated assessment tools and the detailed characterization of clinical populations represented important strengths. Nevertheless, common limitations included the predominance of cross-sectional designs, reliance on self-reported dietary and sexual function measures, limited evidence from general population samples, and insufficient control of potential confounding variables such as psychological status, partner relationships, medication use, and sociocultural factors. Overall, the available evidence suggests that higher adherence to the Mediterranean diet may be associated with better sexual health outcomes, particularly among women with metabolic or chronic inflammatory conditions. However, larger and well-designed randomized controlled trials are needed to strengthen causal inference.

### Thematic overview of findings

The findings of the included studies were synthesized into 4 interrelated themes:

- Mediterranean diet adherence and female sexual function
- Mediterranean diet in clinical and disease-specific populations
- Symptom burden, pain, and sexual well-being
- Potential mechanisms linking the Mediterranean diet and female sexual health

Overall, the included studies generally suggest that greater adherence to the Mediterranean diet is associated with better female sexual function, improved sexual well-being, and a lower prevalence of FSD. However, the strength and consistency of these associations vary across different clinical populations, including women with type 2 diabetes, obesity, inflammatory bowel disease, chronic liver disease, and endometriosis.^15–20^

Furthermore, the available evidence indicates that the relationship between the Mediterranean diet and female sexual health extends beyond improvements in sexual function alone. Potential benefits appear to be mediated through interconnected biological and psychosocial mechanisms, including improved metabolic and vascular health, reduced inflammation and oxidative stress, enhanced psychological well-being, and alleviation of symptom burden.^18^^,^^20^ Accordingly, the 4 themes presented below should be interpreted as complementary and interconnected domains that collectively describe the current evidence rather than as independent categories.

### Theme 1. Mediterranean diet adherence and female sexual function

Most included studies reported that greater adherence to the Mediterranean diet was associated with improved female sexual function and a lower prevalence of FSD. Specifically, among women with type 2 diabetes, higher adherence to the Mediterranean diet was associated with lower FSD prevalence, higher FSFI scores, and a greater proportion of sexually active women.^15^ Similarly, the randomized controlled MÈDITA trial demonstrated that adherence to a Mediterranean dietary pattern attenuated the deterioration of female sexual function over time compared with a low-fat diet, providing longitudinal evidence supporting this association.^16^

### Sexual function domains

Several studies also evaluated individual domains of female sexual function rather than overall FSFI scores alone. The available evidence suggests that the observed associations may be more pronounced for specific domains, particularly lubrication, orgasm, and sexual satisfaction. However, findings across studies were not entirely consistent. For example, Giugliano et al.^15^ reported statistically significant improvements primarily in the satisfaction domain, whereas other domains showed less consistent associations. These findings suggest that the influence of the Mediterranean diet may differ across dimensions of female sexual function and may vary according to study population and clinical context.

### Theme 2. Mediterranean diet in clinical and disease-specific populations

Most available evidence has been generated in women with specific clinical conditions, including type 2 diabetes, obesity, IBD, chronic liver disease, and endometriosis.^15–20^

Among women with type 2 diabetes, greater adherence to the Mediterranean diet was associated with improved glycemic control, lower prevalence of depression, and reduced rates of FSD.^15^ Likewise, women with severe obesity who adhered more closely to the Mediterranean diet after bariatric surgery demonstrated greater improvements in lubrication, orgasm, and sexual satisfaction than those with lower adherence.^17^

In women with inflammatory bowel disease, adherence to the Mediterranean diet was independently associated with lower odds of FSD, with higher Mediterranean Diet Scores associated with a significantly lower probability of FSD (OR = 0.80, 95% CI 0.653-0.974).^18^ Conversely, among women with chronic liver disease, the initially observed associations between Mediterranean diet adherence and sexual function were no longer significant after multivariable adjustment, suggesting that additional metabolic and psychosocial factors may influence these relationships.^19^

### Theme 3. Symptom burden, pain, and sexual well-being

Beyond conventional measures of female sexual function, several studies examined the relationship between the Mediterranean diet and symptom burden, pain, and overall sexual well-being.

Among women with endometriosis, adherence to the Mediterranean diet was associated with significant reductions in dyspareunia and chronic pelvic pain following dietary intervention.^20^ These improvements may indirectly enhance sexual well-being by reducing pain during sexual activity and improving overall quality of life.

Similarly, the available literature suggests that the benefits of the Mediterranean diet extend beyond sexual function itself and encompass broader patient-reported outcomes, including physical symptoms, pain burden, and health-related quality of life. These findings indicate that improvements in female sexual health may occur through multiple interrelated pathways rather than exclusively through direct effects on sexual function.

### Theme 4. Potential mechanisms linking the Mediterranean diet and female sexual health

The available evidence suggests that the association between the Mediterranean diet and female sexual health may be explained through several interconnected biological and psychosocial mechanisms.

### Metabolic and cardiovascular pathways

Improved glycemic control, healthier lipid profiles, reduced obesity, and better metabolic health have consistently been associated with improved female sexual function.^15^^,^^16^ Factors including HbA1c levels, abdominal obesity, and physical activity appear to contribute substantially to these associations, particularly among women with type 2 diabetes.

### Inflammation and oxidative stress

Studies involving women with endometriosis suggest that the Mediterranean diet may reduce pain and improve sexual well-being through its anti-inflammatory and antioxidant properties.^20^ Greater dietary adherence has been associated with lower oxidative stress and reduced reactive oxygen species production, supporting inflammation as a plausible biological pathway.

### Psychological well-being and quality of life

Psychological well-being represents another important mechanism linking the Mediterranean diet and female sexual health. Studies conducted among women with inflammatory bowel disease and chronic liver disease demonstrated significant associations between sexual function and quality-of-life measures, including the Mental Component Summary and Physical Component Summary scores.^19^ These findings suggest that the Mediterranean diet may improve female sexual health indirectly through its beneficial effects on psychological well-being and overall quality of life.

### Quality assessment

In this scoping review, quality assessment was conducted not as a criterion for study exclusion but to facilitate the interpretation of findings within their methodological context. The methodological characteristics and potential limitations of the included studies were evaluated using the Mixed Methods Appraisal Tool (MMAT), which enables the appraisal of diverse research designs (Table 4).

**Table 4:** Methodological Quality Appraisal of Included Studies Using the Mixed Methods Appraisal Tool (MMAT).

**Study**	**Design**	MMAT criteria met^*^	**Overall appraisal**	**Key methodological comments**
Giugliano et al.^15^	Cross-sectional study	4/5	High	Large sample of women with type 2 diabetes and validated FSFI assessment; observational design limits causal inference.
Maiorino et al.^16^	Randomized controlled trial (MÈDITA trial)	5/5	High	Strong long-term randomized design with comparison group and standardized Mediterranean diet intervention.
Ruiz-Tovar et al.^17^	Retrospective observational study	4/5	High	Clinically relevant bariatric population; validated FSFI and PREDIMED assessment, although retrospective design limits causal interpretation.
Romano et al.^18^	Cross-sectional observational study	4/5	High	Disease-specific female cohort with validated FSFI and Mediterranean diet adherence measures; limited generalizability beyond IBD populations.
Romano et al.^19^	Cross-sectional observational study	4/5	High	Comprehensive assessment of sexual function and Mediterranean diet adherence; observational design precludes causal conclusions.
Cirillo et al.^20^	Prospective intervention study	3/5	Moderate	Longitudinal Mediterranean diet intervention in women with endometriosis; focused primarily on dyspareunia and pain outcomes rather than multidimensional sexual function.
Ott et al.^21^	Observational intervention study	3/5	Moderate	Relevant endometriosis population and Mediterranean diet intervention; lacked validated multidimensional sexual function measures such as FSFI.

Abbreviations: FSFI, Female Sexual Function Index; IBD, inflammatory bowel disease; VAS, Visual Analog Scale.

Overall, most included studies employed cross-sectional observational designs, while only a limited number of prospective intervention studies and randomized controlled trials were identified. The MÈDITA randomized controlled trial conducted among individuals with type 2 diabetes met the MMAT criteria for randomized controlled trials (appropriate randomization, comparable groups at baseline, complete outcome data, and adherence to the assigned intervention); its long follow-up period and standardized outcome measures further supported this appraisal.^16^ Similarly, the prospective intervention study involving women with endometriosis provided important methodological strengths through longitudinal follow-up and assessment of dietary adherence over time.^20^

Most studies used validated instruments to assess female sexual function, particularly the FSFI. Studies conducted among women with diabetes, IBD, chronic liver disease, and obesity met most MMAT criteria for quantitative descriptive studies (relevant sampling strategy, representative sample, appropriate measurements, and appropriate statistical analysis), although the risk of non-response bias remained unclear in several studies.^15^^,^^17^^,^^20^ Nevertheless, reliance on self-reported assessments for both dietary adherence and sexual function outcomes represents a potential source of measurement bias.

Several common methodological limitations were identified across studies. The predominance of cross-sectional designs restricts causal inference, while relatively small sample sizes and the frequent focus on specific clinical populations may limit the generalizability of findings to the broader female population. In addition, important confounders were not consistently controlled across studies.

Considerable methodological heterogeneity was also observed regarding outcome assessment. While some studies directly evaluated female sexual function using validated sexual function measures,^15^^,^^16^ others focused on symptom burden, pain outcomes, or quality of life indicators.^20^^,^^22^ This heterogeneity limited direct comparison of findings across studies.

No study was excluded on the basis of methodological quality. Consistent with the exploratory and inclusive nature of scoping review methodology, quality assessment findings were used to support a cautious and contextual interpretation of the evidence synthesized in the thematic analysis.

## Discussion

This scoping review systematically mapped the current evidence on the relationship between adherence to the Mediterranean diet and female sexual health. Overall, greater adherence to the Mediterranean diet was associated with better female sexual function and sexual well-being, particularly among women with diabetes, obesity, IBD, endometriosis, and chronic liver disease.^12^^,^^15–21^^,^^23–26^ These findings are compatible with the hypothesis that the Mediterranean diet may be associated with female sexual health through interconnected metabolic, vascular, inflammatory, and psychosocial mechanisms.

One of the most consistent findings was the association between higher Mediterranean diet adherence and lower rates of FSD, particularly among women with type 2 diabetes.^15^^,^^16^ Given the established role of endothelial dysfunction, vascular impairment, oxidative stress, and chronic inflammation in diabetes-related sexual dysfunction, these findings are biologically plausible. The MÈDITA randomized controlled trial further strengthens this evidence by suggesting that adherence to the Mediterranean diet may reduce the progression of sexual dysfunction over time rather than simply being associated with current sexual function.^12^

The mapped evidence also suggests that associations may be more apparent for lubrication, orgasm, and sexual satisfaction than for sexual desire; however, as this pattern derives from a small number of observational studies, it should be regarded as a hypothesis to be tested rather than an established effect.^17^ This pattern is consistent with the vascular mechanisms underlying genital arousal, whereas sexual desire is more strongly influenced by hormonal, psychological, relational, and sociocultural factors. Beneficial associations were particularly evident among women with inflammatory and metabolic disorders. Greater adherence to the Mediterranean diet was associated with better sexual function after bariatric surgery,^17^ reduced risk of FSD in women with IBD,^18^ and significant improvements in dyspareunia and pelvic pain among women with endometriosis.^20^ These findings support the hypothesis that reducing systemic inflammation and oxidative stress may represent an important pathway linking Mediterranean dietary patterns with female sexual health.

Psychological well-being may also contribute to this relationship. Several studies reported positive associations between sexual function and quality-of-life measures,^19^ while recent evidence suggests that healthier dietary patterns may improve both psychological well-being and female sexual function.^9^^,^^19^^,^^21–23^ In women with chronic liver disease, Mediterranean diet adherence was associated with better quality-of-life indicators, although sexual function remained influenced by broader biopsychosocial factors.^20^^,^^24–26^

Despite these encouraging findings, the available evidence has important methodological limitations. Most studies were cross-sectional, preventing causal inference, and both dietary adherence and sexual function were primarily assessed using self-report instruments. Considerable heterogeneity in study populations, dietary assessment methods, outcome measures, and adjustment for confounding factors—including menopausal status, hormone therapy, depression, physical activity, partner relationship, and socioeconomic status—also limits direct comparison across studies. In particular, Mediterranean diet adherence was assessed with different indices (eg, MEDAS, Prevention with Mediterranean Diet (PREDIMED) -based scores, and Mediterranean Diet Score), whereas sexual health was assessed mainly with the FSFI but also with pain and quality-of-life measures; identical scores therefore do not necessarily represent identical exposure or outcome constructs, which markedly restricts comparability across studies.

From a clinical perspective, the findings support considering the Mediterranean diet as part of a comprehensive lifestyle approach to promote female sexual health, particularly among women with metabolic or inflammatory conditions. However, Mediterranean diet counseling should be regarded as a complementary lifestyle approach that supports, rather than replaces, established biopsychosocial management strategies, and not as a stand-alone intervention for FSD. Future research should prioritize adequately powered randomized controlled trials with standardized dietary and sexual function assessments, longer follow-up periods, standardized dietary assessment methods, validated female sexual health outcome measures, and more diverse populations to clarify causal mechanisms and determine whether improvements in metabolic and inflammatory markers directly mediate changes in female sexual function.

Emerging evidence also suggests that the beneficial effects of the Mediterranean diet may extend to female sexual and genitourinary health through modulation of the gut microbiota and preservation of intestinal barrier integrity. The Mediterranean dietary pattern promotes a diverse and metabolically favorable intestinal microbiota, increases the production of short-chain fatty acids, and contributes to improved intestinal permeability. These changes may reduce systemic inflammation and immune activation, mechanisms that have been implicated in the maintenance of genitourinary tract homeostasis. Alterations in the gut microbiota and disruption of intestinal barrier function have been proposed as potential contributors to urogenital disorders through immune, inflammatory, and microbiota-mediated pathways. Therefore, the gut microbiota–gut barrier axis represents a plausible additional mechanism linking adherence to the Mediterranean diet with improved female sexual health, although direct clinical evidence remains limited and further longitudinal and mechanistic studies are needed to clarify this relationship.^27^ It should be emphasized that none of the 7 included studies examined the gut microbiota or intestinal permeability; this pathway is therefore presented as a theoretical mechanism derived from supporting literature rather than as a finding of the present review.

### Limitations and strengths

A major strength of this review is that it provides one of the most comprehensive syntheses of the association between the Mediterranean diet and female sexual health across metabolic, inflammatory, psychological, and gynecological conditions. By including diverse clinical populations, it highlights female sexual health as a multidimensional outcome influenced by vascular, metabolic, inflammatory, and psychosocial mechanisms rather than hormonal or psychological factors alone.

However, several limitations should be acknowledged. Most included studies were cross-sectional, limiting causal inference. Considerable heterogeneity in study populations, dietary assessment methods, and sexual function measures reduced comparability across studies. Most outcomes were based on self-report instruments, increasing the risk of reporting bias, while important confounders were not consistently controlled. In addition, evidence largely originated from clinical populations and Mediterranean countries, limiting generalizability to healthy women and other cultural settings. Finally, as a scoping review, this study did not quantitatively synthesize the available evidence.

## Conclusion

Within a limited and methodologically heterogeneous evidence base derived mainly from specific clinical populations, greater adherence to the Mediterranean diet was generally associated with better female sexual function and sexual well-being, particularly among women with diabetes, obesity, inflammatory bowel disease, and endometriosis. Improvements in metabolic health, vascular function, inflammation, and psychological well-being have been proposed as possible explanatory pathways; however, these mechanisms were not directly tested in the included studies, and the predominance of cross-sectional designs precludes causal inference.

From a clinical perspective, nutritional counseling based on the Mediterranean diet may be considered a complementary component of the multidisciplinary management of FSD rather than a stand-alone treatment; given the low certainty of the current evidence, this suggestion should be regarded as tentative. However, given the predominance of cross-sectional studies and methodological heterogeneity, these findings should be interpreted cautiously. Future well-designed prospective studies and randomized controlled trials incorporating biological markers, standardized dietary assessment methods, and validated female sexual health outcome measures are needed to clarify causal mechanisms and strengthen the evidence base.

## Data Availability

The datasets used and/or analyzed during the current study are available from the corresponding author upon reasonable request.
